# A systematic review of clinical, epidemiological and demographic predictors of tuberculosis in children with pneumonia

**DOI:** 10.7189/jogh.12.10010

**Published:** 2022-08-09

**Authors:** Saniya Kazi, Hannah Corcoran, Yara-Natalie Abo, Hamish Graham, Jacquie Oliwa, Stephen M Graham, the ARI Review group

**Affiliations:** 1 Murdoch Children’s Research Institute, Melbourne, Victoria, Australia; 2 Royal Children’s Hospital Melbourne, Melbourne, Victoria, Australia; 3 Monash Health, Melbourne, Victoria, Australia; 4 University of Melbourne Department of Paediatrics, Melbourne, Victoria, Australia; 5 KEMRI-Wellcome Trust Research Programme, Nairobi, Kenya; 6 University of Nairobi, Nairobi, Kenya

## Abstract

**Background:**

Tuberculosis (TB) can present as acute, severe pneumonia in children, but features which distinguish TB from other causes of pneumonia are not well understood. We conducted a systematic review to determine the prevalence and to explore clinical and demographic predictors of TB in children presenting with pneumonia over three decades.

**Methods:**

We searched for peer-reviewed, English language studies published between 1990 and 2020 that included children aged between 1 month and 17 years with pneumonia and prospectively evaluated for TB. There were 895 abstracts and titles screened, and 72 full text articles assessed for eligibility.

**Results:**

Thirteen clinical studies, two autopsy studies and one systematic review were included in analyses. Majority of studies were from Africa (12/15) and included children less than five years age. Prevalence of bacteriologically confirmed TB in children with pneumonia ranged from 0.2% to 14.8% (median = 3.7%, interquartile range (IQR) = 5.95) and remained stable over the three decades. TB may be more likely in children with pneumonia if they have a history of close TB contact, HIV infection, malnutrition, age less than one year or failure to respond to empirical antibiotics. However, these features have limited discriminatory value as TB commonly presents as acute severe pneumonia – with a short duration of cough, and clinical and radiographic features indistinguishable from other causes of pneumonia. Approximately half of patients with TB respond to initial empirical antibiotics, presumably due to bacterial co-infection, and follow-up may be critical to detect and treat TB.

**Conclusion:**

TB should be considered as a potential cause or comorbidity in all children presenting with pneumonia in high burden settings. Clinicians should be alert to the presence of known risk factors for TB and bacteriological confirmation sought whenever possible. Quality data regarding clinical predictors of TB in childhood pneumonia are lacking. Further, prospective research is needed to better understand predictors and prevalence of TB in childhood pneumonia, particularly in TB endemic settings outside of Africa and in older children. Children of all ages with pneumonia should be included in research on improved, point-of-care TB diagnostics to support early case detection and treatment.

Pneumonia is a major cause of mortality in young children, accounting for 15% of global under-5 mortality in 2014 [1]. *Mycobacterium tuberculosis* is recognized as a primary cause of, or contributor to, pneumonia presentations in children in tuberculosis (TB) endemic settings [2]. The potential importance of TB in this context is further enhanced as the burden of other bacterial causes of pneumonia declines with increased vaccine coverage [3]. TB treatment is safe and effective, with excellent outcomes in the majority of children. Child-friendly dispersible fixed-dose formulations are now widely available and have improved the feasibility of treating young children for TB [4].

Improving pneumonia outcomes in children with TB requires prompt initiation of treatment and this is dependent upon early case detection. However, a significant gap remains in TB detection, particularly in young children and those presenting atypically such as with acute pneumonia [2,5,6]. This is highlighted by findings from autopsy studies in high TB incidence settings that have identified undiagnosed TB in children who have died of pneumonia [2]. Bacteriological confirmation of TB diagnosis through culture or rapid molecular diagnostics has low sensitivity, and is impacted by patient age, comorbidities, disease stage as well as laboratory technique [7]. Furthermore, access to diagnostic facilities may be limited at primary and secondary levels of care [8]. Therefore, identifying TB clinically, remains pivotal.

The primary objective of this study was to systematically review the literature to assess the clinical signs, symptoms, and demographic features that predict TB disease in children with pneumonia. We secondarily evaluated the prevalence of TB in children with pneumonia in high TB burden regions, over the previous three decades.

## METHODS

This study was conducted following the Preferred Reporting Items for Systematic Reviews and Meta-Analyses (PRISMA) statement. The study protocol was registered in the Prospective Register of Systematic Reviews (PROSPERO) on 31 October 2020 (PROSPERO ID: CRD42020206895).

### Data sources

We searched MEDLINE, EMBASE, PubMed and Global Index Medicus databases for English-language studies published from January 1, 1990 to August 30, 2020. References of included studies were also screened. The search strategy is detailed in Appendix S1 in the **Online Supplementary Document**.

### Study selection and eligibility criteria

Original studies were included if they recruited a minimum of 50 children aged 1 month-17 years with pneumonia and reported clinical or demographic features in children with bacteriologically confirmed TB and those without TB, or reported prevalence of TB within the study population. Studies were excluded if: extraction of data for the defined age range was not possible; pneumonia or acute respiratory infection was not the entry point; the study was not related to clinical or demographic predictors of TB or did not report TB prevalence; bacteriological confirmation of TB was not attempted; or the study provided insufficient detail regarding TB diagnostic criteria. Narrative reviews, case reports, conference abstracts and opinion articles were also excluded.

### Data extraction and synthesis

The Covidence® platform was utilised for record management from screening to data extraction stages. Two reviewers independently assessed articles for relevance, and discrepancies were resolved by discussion, including a third reviewer where necessary. Full text studies were evaluated for eligibility by two reviewers and data were extracted using a custom template. Study quality was assessed using a standardised tool, the Effective Public Health Practice Project (EPHPP) Quality Assessment Tool for Quantitative Studies [9]. Findings from a previous systematic review [2] informed our narrative approach to presenting the data, and we did not conduct a formal meta-analysis as we anticipated heterogeneity in study population, study design, and diagnostic methods. Rather we present the individual study results and provide summary prevalence estimates (median and interquartile range) for diagnostic categories (eg, bacteriologically confirmed, clinical diagnosis).

## RESULTS

### Study selection

**Figure 1:**
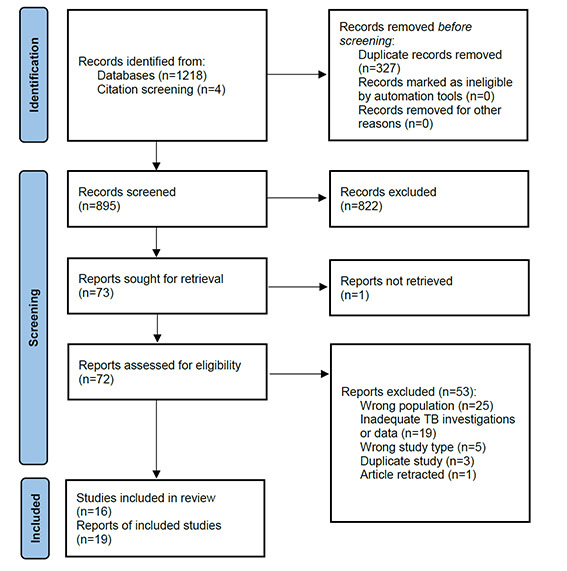
PRISMA 2020 flow diagram of literature search.

891 unique citations were identified through database searches, four additional studies were identified through reference screening and 72 full text articles were assessed for eligibility, as demonstrated in **Figure 1****.** Thirteen clinical studies and two autopsy studies, presented in **Table 1****,** as well as one systematic review [2] met eligibility criteria. Three additional studies provided supplementary data related to an original study included in the analysis [25-27].

**Table 1:** Summary of study characteristics

Study	Country	Study design	Participants	Population description	Diagnostic method	Quality†
Prospective clinical studies						
Adegbola [10], 1994	The Gambia	Prospective cohort study	n = 278, 3-59 mo	Malnourished children with clinical and radiologic pneumonia	Microscopy + culture on lung aspirate or IS when TB suspected	Moderate
Bolursaz [11], 2017	Iran	Cross-sectional study	n = 229, 1 mo18 y old (mean 96 mo)	Clinical and radiologic (lobar or bronchopneumonic infiltration) pneumonia	Positive microbiological tests for TB (sputum, GA or broncho-alveolar lavage), unclear threshold for testing	Weak
Chisti [12], 2015	Bangladesh	Prospective case-control study	n = 405, 0-59 mo (median 10 mo)	Severely malnourished children with cough and/or respiratory distress and radiologic pneumonia	Culture or Xpert MTB/RIF of sputum	Moderate
Graham[13], 2011	Malawi	Prospective cohort study	n = 327, 2 mo-14 y (median 5 mo)	Severe or Very Severe Pneumonia (WHO, 2005)	IS microscopy and culture when TB suspected	Weak
Hammitt [14], 2012	Kenya	Prospective case-control study	n = 810, 2-59 mo	Severe or Very Severe Pneumonia (WHO, 2005)	IS microscopy and culture when TB suspected	Moderate
Madhi [15], 2000	South Africa	Prospective cohort study	n = 1215, 2-59 mo	WHO Severe Lower Respiratory Tract Infection (WHO, 1990) and/or oxygen saturation <100%	Microscopy and culture of GA at physician discretion	Weak
McNally [16], 2007	South Africa	Prospective cohort study	n = 358, 1-59 mo (mean 4.8 mo)	Severe or Very Severe Pneumonia (WHO, 1990)	Microscopy and culture of IS and GA +/− BAL if treatment failure at 48 h	Strong
Moore [17], 2010	South Africa	Post hoc analysis of a Randomised Controlled Trial	n = 2439 (hospital admissions), 2 mo-18 y old	Study participants of PCV9 vaccine probe study hospitalised with LRTI	Culture of sputum when TB suspected	Weak
Moore [18], 2017	South Africa	Prospective cohort study	n = 920, 1-59 mo	Severe or Very Severe Pneumonia (WHO, 2005)	Culture of IS, GA or both, +/− ETT samples	Moderate
Nantongo [19], 2013	Uganda	Cross-sectional study	n = 270, 2 mo-12 y (median 15months)	Severe or Very Severe Pneumonia (WHO, 2005)	Culture of blood and sputum	Strong
O'Brien [20], 2019	Multiple*	Prospective case-control study	n = 1769, 1-59 mo	Severe or Very Severe Pneumonia (WHO, 2005) and radiologic pneumonia in HIV uninfected children	Culture of IS or GA	Strong
Uriyo [21 2006	Tanzania	Prospective cohort study	n = 72, 1-59 mo (mean 13.5 mo)	LRTI (WHO, 2001)	DNA amplification assay on IS (culture unavailable)	Weak
Zar[22] 2001	South Africa	Prospective cohort study	n = 250, 1 mo-18 y (mean 6 mo)	Pneumonia (WHO, 1990)	Microscopy and culture of IS, GA +/− BAL	Strong
Post-mortem studies:						
Chintu [23], 2002	Zambia	Necropsy study	n = 264, 1 mo-16 y old	Post-mortem of HIV-uninfected and HIV-infected children hospitalised with respiratory symptoms	AFB smear of lung tissue + findings of typical necrosis on lung autopsy	Moderate
Rennert [24], 2002	South Africa	Necropsy study	n = 93, 1 mo-18 y (mean 10.5 mo)	Post-mortem of HIV-infected children hospitalised with clinical or radiologic lung disease	AFB smear, culture and histology of lung tissue from point biopsies	Moderate

AFB – acid fast bacilli, BAL – bronchoalveolar lavage, ETT – endotracheal tube, GA – gastric aspirate, IS – induced sputum

*Included sites in Bangladesh, The Gambia, Kenya, Mali, South Africa, Thailand and Zambia.

†EPHPP Quality Assessment Tool Global Rating Score, at outcome level.

### Study characteristics

Ten of 13 clinical studies were prospective in design, two were cross-sectional studies and one was a post-hoc analysis of a multi-country RCT. Eight of the clinical studies were in children with severe or very severe pneumonia and two specifically evaluated severely malnourished children [10,12]. The majority of studies (10/16) were from high TB burden settings (incidence >299 per 100 000 per year). Most studies were from the African region (12/15), one multi-country study comprised study populations over eight countries in Africa and Asia, and the remaining studies were conducted in Bangladesh and Iran. One large study excluded children with HIV-infection in the primary analysis [20], while the studies in African populations included children with and without HIV infection (**Table 2**).

**Table 2:** Prevalence of TB in children with pneumonia

Author Country of study	Study year	Population TB incidence*	Investigated for TB/Participants (%)	Bacteriologically Confirmed n (%)	Clinically diagnosed n (%)†	All TB diagnoses n (%)	HIV seroprevalence in participants (%)	Co-infections and/or TB case fatality rate
Adegbola [10] The Gambia	1990-92	189/100 000	?/278	4 (1.4%)	-	4 (1.4%)	1.9%	
Madhi [15] South Africa	1997-98	406/100 000	858/1215 (70.6%)	69 (5.7%)	-	69 (5.7%)	45.1%	10% concurrent bacteraemia
Zar [22] South Africa	1998	406/100 000	210/250 (84%)	20 (8.0%)	-	20 (8.0%)	60.4%	TB case fatality rate: 15%
Moore [17] South Africa	1998-2006	746/100 000	1334/2439^‡^ (54.7%)	90 (3.7%)	326 (13.3%)	416 (17.1%)	63.9%	*S Pneumoniae* in 17/416 TB diagnoses (1/90 bacteriologically confirmed); TB case fatality: 4%
McNally [16] South Africa	2001-02	666/100 000	358/358 (100%)	53 (14.8%)	-	53 (14.8%)	67.6%	4/53: 1 *S pneumoniae* bacteraemia, 3 organisms in pleural fluid; TB case fatality rate: 20.7%
Uriyo [21] Tanzania	2003	344/100 000	72/72 (100%)	1 (1.4%)	-	1 (1.4%)	30.6%	*S pneumoniae* in 1/1
Graham [13] Malawi	2005-06	398/100 000	?/327	1 (0.3%)	9 (2.8%)	10 (3.1%)	41%	3/10: 2 *S pneumoniae*, 1 *H influenzae*
Hammitt [14] Kenya	2010	531/100 000	108/810 (13.3%)	2 (0.2%)	3 (0.4%)	5 (0.6%)	8.5%	
Nantongo [19] Uganda	2011	207 100 000	270/270 (100%)	17 (6.3%)	34 (12.6%)	51 (18.9%)	15%	
Chisti [12] Bangladesh	2011-12	225/100 000	405/405 (100%)	27 (6.7%)	60 (14.8%)	87 (21.5%)	Not tested	11% TB cases died at home whilst receiving TB treatment
Moore [18] South Africa	2011-13	922/100 000	906/920 (98.5%)	27 (2.9%)	119 (12.9%)	146 (15.9%)	32.9%	
O'Brien [20] Multiple	2011-2014	61-892 /10, 000	1571/1769 (88.5%)	31 (1.4%)	-	31 (1.4%)	Excluded	
Bolursaz [11] Iran	2013	18/100 000	?/229^§^	29 (12.7%)	2 (0.9%)	31 (13.5%)	Not reported	
Post-mortem studies								
Chintu [23] Zambia	1997-2000	759/100 000	264/264 (100%)	54 (20.5%)	-	54 (20.5%)	68%	
Rennert [24] South Africa	1998-99	406/100 000	93 (100%)	4 (4.3%)	17 antemortem diagnoses	21 (22.6%)	100%	0/4 with TB on autopsy had other pathogens identified

*Data obtained from The World Bank [28] to estimate TB incidence at mid-point of study.

^†^Definition of clinically diagnosed TB varied between studies.

^‡^Number refers to pneumonia admission episodes; number of participants not provided.

^§^Included 101 children with persistent pneumonia and 128 children with recurrent pneumonia.

Only eight studies compared clinical or demographic features in children with a diagnosis of TB to those without [10,12,15-17,19,20,22]. Prevalence of TB, a secondary outcome, was reported in 13 clinical studies and two necropsy studies [23,24] (see **Table 2**). All studies were required to have sought bacteriological confirmation of TB, for inclusion in review, however five studies obtained samples for *M tuberculosis* only when TB was clinically suspected [10,13-15,17]. Studies were highly heterogeneous in diagnostic approach to TB. The majority of specimens obtained for microbiological culture were sputum, obtained through gastric aspirate or sputum induction. Only one study performed Xpert MTB/RIF (Cepheid, CA, USA) [12].

One systematic review by Oliwa et al [2] explored the burden of TB in child pneumonia in studies published from 1949 to 2014. Fourteen studies were included, 11 of which also contributed to our systematic review, while three studies did not meet our eligibility criteria due to the study population or inadequate TB work up.

### Quality assessment

The quality of studies with respect to our study outcomes, assessed using the EPHPP tool, are presented in **Table 1** and Appendix S3 in the **Online Supplementary Document**. Five studies were assessed as being of weak quality, six studies were considered moderate quality [10-12,14,23,24] and the remaining four studies were strong in quality. Studies which were considered weak in quality, did not perform adequate diagnostic work up for TB on all patients, with potential for confounding bias in reporting of clinical predictors of TB and underestimation of TB prevalence. Eight studies provided data regarding clinical predictors of TB [10,12,15-17,19,20,22], however only two studies were conducted with evaluation of TB features as a primary aim [12,16]. Older children were poorly represented in studies, with only three studies including children up to 17 years of age [17,21,22]. Some studies evaluated children with a comorbidity, such as HIV infection [24] or malnutrition [10,12]. All studies provided important data that was applicable to our target population and research questions, within the limitations described.

### Prevalence of tuberculosis in children presenting with pneumonia

Prevalence of bacteriologically confirmed and clinically diagnosed TB in individual studies is presented in **Table 2****.** The prevalence of bacteriologically confirmed TB varied widely (range = 0.2%-14.8%, median = 3.7%, interquartile range [IQR] = 5.95), reflecting heterogeneity in study settings, populations, diagnostic approach to TB and local TB epidemiology. Eight studies attempted bacteriological confirmation for TB in all pneumonia patients, however diagnostic methods were often suboptimal due to resource challenges. Two clinical studies which identified bacteriologically confirmed TB in over one in 10 children with pneumonia focused on children not responding to initial pneumonia treatment [16,21]. Clinically diagnosed TB cases were reported in seven studies (range = 0.4%-14.8%, median = 12.6%, IQR = 12.4), however diagnostic definitions varied greatly. Two autopsy studies in high TB and HIV burden settings, identified TB in 4.3% (4/93, Zambia) and 20.5% (54/264, South Africa) of children who died of pneumonia [23,24].

Despite changes in pneumonia epidemiology over time, TB has remained prevalent in children with pneumonia (Figure S2 in the **Online Supplementary Document**). The PERCH study [20] evaluated aetiology of severe pneumonia in HIV-uninfected children with radiological lobar pneumonia, in seven low-income and lower-middle income countries. Culture-confirmed TB was diagnosed in 1.5% of severe and very severe pneumonia cases, based on one induced sputum or gastric lavage sample. A second sputum specimen resulted in a 50% increase in yield and TB prevalence of 2.9%, in a sub study conducted in a South African site of the PERCH study [18]. The PERCH study applied modelling (Bayesian, partial latent class analysis) to estimate the true probability of aetiological agents based on known sensitivities of laboratory tests, and individual and population data and TB was estimated to account for 5.9% (3.9-8.3) of severe pneumonia cases with this modelling.

### Predictors of tuberculosis in children presenting with pneumonia

The demographic, epidemiological and clinical features that were reported in children with pneumonia, with and without bacteriologically confirmed TB, are reported in **Table 3****.** Eight studies contributed to these results [10,12,15-17,19,20,22].

**Table 3:** Clinical and demographic features in children with bacteriologically confirmed tuberculosis presenting with pneumonia

Study	TB Contact History	Comorbidities	Age	Clinical Features
Adegbola [10] 1994		Malnourished children with pneumonia more likely to have TB.		All children with bacteriologically confirmed TB in this study were malnourished. However, malnourished children were more likely to be investigated for TB in this study
Chisti [12] 2015	History of active TB contact: 19% of children with bacteriologically confirmed TB: 13% with clinically diagnosed TB and 0.3% with no TB, RR 4.69 (95% CI = 3.23-6.78)	This study evaluated children with severe acute malnutrition only 27/405 children had bacteriologically confirmed TB and a further 60/405 children had clinically diagnosed TB		All children in this study had acute symptoms (<2 weeks duration) Mean duration of fever higher in cases with TB vs non-TB (6.5 d vs 4.0 d) Chest x-ray findings were similar in children with and without TB; however, CXR consolidation formed part of inclusion criteria	
Madhi [15] 2000		RR of 22.5 (95% CI = 13.45-37.62) for having severe pneumonia and bacteriologically confirmed TB in HIV infected vs HIV uninfected children (incidence 1470 vs 65/100 000).	83.9% of children with bacteriologically confirmed TB presenting with severe pneumonia were <1 y age. Median age of children diagnosed with bacteriologically confirmed TB did not differ by HIV status.	Nutritional status of children with bacteriologically confirmed TB did not differ by HIV status.	
McNally [16] 2007	Maternal history of TB was associated with treatment failure and mortality in children with pneumonia, but not with bacteriologically confirmed TB.	Prevalence of bacteriologically confirmed TB in children presenting with pneumonia similar in children with HIV infection and without.	No association between age and diagnosis of bacteriologically confirmed TB	Response to initial pneumonia treatment: 24/53 (45.3%) with bacteriologically confirmed TB failed to respond to treatment at 48 h. 24/110 (21.8%) who failed empirical pneumonia treatment at 48 h were diagnosed with bacteriologically confirmed TB. Prolonged cough (>2 weeks duration) was only present in 15% of children with bacteriologically confirmed TB	
Moore [17] 2010				Median cough duration in children with bacteriologically confirmed TB was 4 d (IQR 2-7). 48.9% (44/90) bacteriologically confirmed TB cases were discharged following response to empirical antibiotics without commencing TB treatment	
Nantongo[19] 2013	History of recent, smear positive TB contact significantly increased likelihood of TB	HIV prevalence higher in children with bacteriologically confirmed TB (27.5% vs 15% in all participants). 34% of the HIV positive children presenting with severe pneumonia had pulmonary TB. Only 1 of the 14 TB cases was on ARVs while 6 without TB were on ARVs.	Age <2 y significantly associated TB in HIV negative children with severe pneumonia and age <5 y significantly associated with TB on multivariate analysis in both HIV positive and HIV negative children	History of cough >2 weeks duration, recent weight loss and significant peripheral lymphadenopathy were associated with TB in children with pneumonia. 33.3% of children with TB vs 15.1% of children without TB had recent weight loss. Abnormal CXRs were found in (45/51) 88% of TB cases vs (136/219) 62% of those without TB OR 4.6 (1.8-11.2), *P* < 0.001. No significant difference in proportion of children with BCG scar (BCG scar absent in 35.3% with TB vs 26.9% without TB)	
O’Brien [20] 2019			TB diagnosis more common in <1 y age: 18/975 (1.8%) than in age>/ = 1year: 6/596 (1%)	Prevalence of TB was higher in children with very severe pneumonia (2.2%) compared with severe pneumonia (1.3%) (WHO 2005 definition)
Zar [22] 2001		No significant difference in bacteriologically confirmed TB prevalence in HIV-positive vs HIV-negative children - 7.9% v 8%,*P* = 0.97. HIV infected children were more likely to have had prior TB treatment (17 v 0) and admission, and were older	Median age with bacteriologically confirmed TB was 12 (7-25) months, similar to those without TB of 9 (3–21.5) months; *P* = 0.19.	Chest x-ray finding of hilar or mediastinal lymphadenopathy was more common in children with TB-43% vs 12.2% in children without TB, *P* = 0.006	
Post-mortem studies				
Rennert [24] 2002	3 (75%) of 4 HIV-infected children with documented TB had adult TB contact vs 1 (1.4%) of 72 children with no TB	HIV infection (CDC category B or C) was a requirement for inclusion in this study	Prevalence of TB in HIV-infected children aged 1 y or older at time of death was 13% (3/23), vs 1.4% (1/70) in children <1 y old (RR 9.1, 95% CI = 1.0-83.5). Older children were more readily treated for suspected TB (30.4% vs 14.5% in younger patients, 95% CI = 0.9-4.9).	There were no significant differences in clinical findings in patients with TB and those without. Of the 4 children with evidence of TB on post-mortem investigations: 0/4 had cough >1 week; 4/4 had fever; 2/4 weighed <60% expected; 3/4 had CDC category C HIV disease, 1/4 had CDC category HIV disease.	

#### Close (household) contact with a confirmed TB case

A history of contact with a sputum smear-positive TB case within one year was significantly associated with bacteriologically confirmed TB in children with severe pneumonia. A necropsy study of children with HIV infection determined this to be the strongest predictor of TB [24]. However, Chisti et al. [12] found a positive TB contact history was only reported in a minority of children (19%) with bacteriologically confirmed TB, indicating that the absence of a declared TB contact has poor negative predictive value. A South African study identified maternal active TB to be an independent risk factor for treatment failure and mortality in children with pneumonia, however, it did not find a direct association between maternal active TB and bacteriologically confirmed TB in the child presenting with pneumonia. The authors hypothesized that undiagnosed TB may have contributed to the increased mortality and treatment failure in these children [16].

#### HIV status

Seroprevalence of HIV varied by geographic region and over time (reported in **Table 2**). Children with HIV were identified to have a significantly higher incidence of culture confirmed TB pneumonia than children without HIV infection (1470 vs 65 cases per 100 000 per year in children less than 2 years old (*P* < 0001; RR = 22.5, 95% CI = 13.45-37.62) [15]. The relative proportion of pneumonia cases attributable to TB in children with and without HIV was found to be similar in three studies [15,16,18]. A study from Uganda identified 34% of HIV infected children presenting with pneumonia had bacteriologically confirmed TB, and prevalence was lower in children on antiretroviral therapy, with only one of seven (14.3%) of this sub-group of children identified to have TB [19]. Four studies in South Africa evaluating childhood pneumonia, conducted during a period of high HIV endemicity, identified a similar seroprevalence of HIV in children with and without TB (HIV seroprevalence 45.1%-67.6% in children with TB, 52%-64% in children without TB). Zar et al. [22] identified that children with HIV with severe pneumonia were likely to be older, have had previous admission and more likely to have previously been treated for TB. Madhi et al. [15] identified that children with HIV-infection are significantly more likely to be evaluated for TB and receive a clinical diagnosis.

#### Nutritional status

Malnutrition (WHO weight for age Z score<-2) was prevalent in children with pneumonia in included studies, however few studies compared nutritional status in cases with and without TB. A study in Bangladesh evaluated 405 severely malnourished children (<5 years) with pneumonia and reported a high prevalence of bacteriologically confirmed TB (6.7%); weight for age Z score did not differ between those with or without TB but the study only included children with severe malnutrition [12]. A case-control study from 1994 evaluating malnourished and well-nourished children with pneumonia found malnutrition, particularly when associated with oedema, was associated with TB [10]. However, well-nourished children were significantly less likely to undergo evaluation for TB in this study; 23 of 26 children in whom TB was suspected, were malnourished.

#### Age

The mean age of study participants was less than 24 months in all but one [11] of the included studies. Eight of the studies only included young children (<5 years) – **Table 1**. While young age is a known risk factor for all-cause pneumonia, TB was disproportionately detected in the youngest age groups. The PERCH study identified that infants (<1 year) were disproportionately diagnosed with bacteriologically confirmed TB compared with children aged 1 year or older: 18/975 (1.8%) vs 6/596 (1.0%) [20]. On multivariate analysis, Nantongo et al. [19] identified a significant association between age less than 5 years and TB diagnosis, in HIV infected and uninfected children. Madhi et al. [15] also reported a disproportionately higher incidence of bacteriologically confirmed TB in infants, regardless of HIV status. Data for older children and adolescents were scarce. Only 3 of 14 clinical studies included children up to 18 years of age and age-stratified observations were not reported [11,17,22].

#### Symptoms – duration and severity

Several studies reported that TB commonly presented as acute, severe pneumonia – with a short duration of cough, and no specific distinguishing features [12,15-17,22,24]. As the studies in this review were evaluating children with acute pneumonia presentations, children with prolonged symptoms may not have been selected for inclusion. Nantongo et al. [19] assessed duration of cough in children with pneumonia and reported that a prolonged cough (greater than 14 days duration) and significant peripheral lymphadenopathy were associated with TB. TB was identified in children with severe and non-severe pneumonia, and studies that compared symptomatology found that clinical features were unable to distinguish pneumonia cases with and without TB. The PERCH multi-site study of pneumonia aetiology in HIV-negative children found the prevalence of culture confirmed TB to be higher in children with very severe pneumonia than with severe pneumonia (2.2% vs 1.3%), using the previous WHO definitions for severity [20].

Rennert et al. [24] conducted autopsies of children with HIV and respiratory disease; the three most common aetiological agents of pneumonia identified on autopsy – Cytomegalovirus (CMV), *Pneumocystis jirovecii* and *M.tuberculosis* - were not able to be distinguished by ante-mortem clinical features such as cough, fever, weight and auscultatory signs.

#### Chest x-ray abnormalities

Chest radiography was routinely performed in most studies. Participants were required to have chest x-ray abnormalities typical of pneumonia for inclusion in four of the studies included in the review, with prevalence of TB ranging from 1.4% to 12.7% [10-12,20]. Two studies compared radiological findings in children with a diagnosis of TB and those without [12,22]. Zar et al. [22] identified hilar or mediastinal adenopathy occurred more commonly in children with TB (43% compared to 12.2% of those without TB, *P* = 0.006). Chisti et al. [12] did not identify discerning features on radiography in TB cases, however this study required participants to have lobar consolidation on chest x-ray for inclusion and looked specifically at children under 5 years of age with severe malnutrition. Rennert et al. [24] conducted post-mortem examinations in HIV-infected children aged 0-6 years old who died of pneumonia and found chest x-ray abnormalities were similar in children with post-mortem diagnosis of TB, pneumocystis pneumonia and CMV.

#### Coinfections

Five studies reported coinfections in bacteriologically confirmed TB and *Streptococcus pneumoniae* was the most common co-pathogen isolated [13,15-17,21]. Results are presented in **Table 2**. All five studies were conducted in the African region, prior to routine pneumococcal immunisation. A post hoc analysis of a pneumococcal conjugate vaccine study in South Africa found that hospitalization for bacteriologically confirmed TB was 43.4% (95% CI = 9.7%-65.1%) less likely among vaccine recipients (n = 30), leading authors to conclude that bacteriologically confirmed TB in this setting was probably associated with superimposed pneumococcal infection [17].

#### Treatment failure

Approximately half of all children with bacteriologically confirmed TB who presented with pneumonia, responded to initial empirical antibiotic treatment [16,17]. Moore et al. [17] reported that 48.9% (44/90) of TB cases responded to empirical antibiotics, and as a result were not initiated on TB treatment, with four of these children dying following discharge. A study conducted in a tertiary referral hospital in Iran, evaluated aetiology of pneumonia in children aged up to 18 years. Bacteriologically confirmed pulmonary TB was diagnosed in 38.75% (29/81) children with persistent pneumonia, defined as continuation of symptoms and radiological abnormalities for more than 30 days despite 10 or more days of antibiotics [21]. McNally et al. [16] did not identify a direct association between bacteriologically confirmed TB and treatment failure, however maternal TB infection conferred a significantly increased risk of treatment failure. 72% (13/18) of cases with a history of maternal TB failed treatment, vs 33% (113/340) of children without maternal history of TB; *P* = 0.0007). Of children who did not respond to empirical antibiotic treatment for pneumonia after 48 hours, TB was the second most common pathogen isolated, after *P.jirovecii*.

#### Mortality

Four studies reported mortality in children with pneumonia and TB [12,16,17,22]. All of these studies were conducted in urban-based hospital settings, between 1990 and 2006. The case fatality rate ranged from 5%-21% (**Table 2**) and bacteriologically confirmed TB was not associated with higher risk of death in children with pneumonia. However, two studies reported increased risk of mortality in children who may have had undetected TB. Moore et al. [17] reported 44 (48.9%) of 90 children with bacteriologically confirmed TB had not received a diagnosis of TB or initiated treatment upon discharge, and 4 (9.1%) of these children died. McNally et al. [16] identified maternal TB infection to be associated with mortality in children with pneumonia. Two necropsy studies performed from 1998-2000 in South Africa (children with HIV infection only) and Zambia (children with and without HIV infection) identified evidence of TB in 4.3 and 20.5% of children who had died with respiratory disease [23,24].

## DISCUSSION

TB is consistently identified as a cause or comorbidity in young children with acute, severe pneumonia in TB-endemic countries. Whilst many other bacterial causes of pneumonia have reduced in prevalence over time [20], the burden of TB in childhood pneumonia has remained significant over three decades. The contribution of TB to pneumonia presentations is likely to be underestimated in included studies, which often have excluded children with radiological features suggestive of TB, recent hospitalisation, or acute-on-chronic symptoms. Moreover, the sensitivity of culture of a single sputum sample for detection of TB is low and impacted by sampling method and laboratory capabilities [29]. Therefore, we estimate *M.tuberculosis* to be a significant pathogen in 5%-10% of children with severe pneumonia presenting in high incidence TB settings.

Limited data from clinical studies suggest that TB, when undetected and untreated, is also associated with pneumonia-related mortality in high-risk populations. This is supported by autopsy studies of children with and without HIV infection, who died in hospital following presentation with respiratory symptoms. A recent autopsy study evaluated all inpatient child deaths in a teaching hospital in Zambia, between 2011 to 2014, and confirmed TB on post-mortem assessment in 10 (8%) of 121 child deaths [29]. Only one child was diagnosed with TB ante-mortem, and the other nine children received physician diagnoses of pneumonia (40%), sepsis or meningitis [29,30]. The unacceptably high post-discharge mortality following inpatient admission with severe pneumonia in high TB burden settings underscores the potential importance of detection and treatment of TB [31].

This review highlights that TB often presents with acute, severe pneumonia that is indistinguishable clinically and radiologically from other pneumonia aetiologies. Bacteriological confirmation should be sought whenever feasible in children with pneumonia suspected to have TB and clinical diagnosis should continue to be made where the index of suspicion is high. A positive history of recent, close contact with a TB case should raise concern about TB, and a contact history should be routinely evaluated in all children presenting with acute severe pneumonia. Young age, prolonged symptoms, and typical radiological findings are also variably associated with TB in children with pneumonia. However, the absence of these features is common in children confirmed to have TB, and hence reliance upon risk factors to alert one to consider TB, is likely to miss a proportion of TB cases. TB prevalence is high amongst children with persistent pneumonia and treatment failure, however approximately half of children with bacteriologically confirmed TB respond to empirical antibiotic treatment for pneumonia, perhaps attributable to co-infections with susceptible pathogens such as *Streptococcus pneumoniae*. Routine follow-up following inpatient admission with pneumonia may provide an opportunity to identify missed cases.

TB disproportionately affects younger children as well as those with HIV infection or severe malnutrition. Given significant advances in HIV treatment over recent decades, and the resulting shift in epidemiology of HIV, there are insufficient data to determine the current relative risk of TB causing pneumonia in children living with HIV infection, compared with those without HIV infection. All studies including children with HIV infection were conducted in the African region. The initial publication of the PERCH study did not include findings in children with HIV infection [20], however additional findings have since been published that include aetiology of severe pneumonia in HIV-positive children [32,33]. *M.tuberculosis* was a common pathogen in HIV-positive Zambian children with WHO-defined severe pneumonia, with an estimated aetiological fraction of 12.8% (95% CI = 4.3-25.3). Both HIV infection and malnutrition increase risk of pneumonia of all causes, and poor nutritional status has a recognized, bi-directional relationship with TB [34]. More research, including from the Asian region, is needed to understand the predictive value of co-morbid malnutrition to identify TB in a child with pneumonia.

This systematic review had several limitations which are common to all included studies. There are limited data evaluating clinical, demographic and epidemiological predictors of TB in children with pneumonia, and little to no data in older children and adolescents. Numbers of children with bacteriologically confirmed TB in most studies were small. Studies included substantial heterogeneity in patient selection and diagnostic methods for bacterial confirmation. The inherent challenge of bacteriological confirmation of TB may impact the assessment of clinical predictors and under-estimate the true burden of TB. Bacteriological confirmation has a higher specificity for TB than clinical diagnosis. However, it may represent a more severe end of the full spectrum of disease due to *M.tuberculosis*.

The WHO has updated and consolidated guidelines on the management of TB in children and adolescents, which include new recommendations relevant to child pneumonia and are accompanied by an operational handbook [35]. Decentralisation of diagnosis and care is supported by practical diagnostic and treatment decision approaches as most children with TB present initially to primary- or secondary-level health facilities [36]. Xpert^®^ MTB/RIF or Ultra (Cepheid, Sunnyvale, CA, USA) is the preferred laboratory diagnostic, and stool or nasopharyngeal aspirate samples are recommended when less invasive alternatives to induced sputum or gastric aspirate are preferred, such as in children with respiratory distress.

Both TB and pneumonia are leading causes of death in young children (<5 years) globally [1,37]. However, while TB is commonly reported in older children and adolescents (5-19 years) in TB endemic countries [38], the contribution of TB to severe pneumonia and pneumonia-related mortality in this age group is not known. Bacteriological confirmation of pulmonary TB has a higher yield in this age group than in young children, and older children and adolescents should be included in future aetiological studies [39]. Many of the WHO high-burden TB countries are in the Asia-Pacific region [40] and the PERCH study recently reported that TB was common in Asian children with severe pneumonia and an abnormal CXR, with a prevalence of 10% and 3.6% in Thailand and Bangladesh respectively [38,41]. However, there is a striking gap in research evaluating TB in childhood pneumonia in global regions other than the African region, including in children with malnutrition or HIV infection.

Future studies should evaluate and report features in children with severe pneumonia that compare those with TB and those without. Many child deaths in pneumonia occur early in the hospital admission, hence there is a need for research evaluating rapid diagnostic techniques, both laboratory-based or clinical, in order to guide early initiation of TB treatment. Future research studies should evaluate non-invasive sputum sampling methods such as stool and nasopharyngeal aspirate and include post-discharge follow-up to better determine the impact of interventions on mortality.

## CONCLUSIONS

*M. tuberculosis* should be considered early as a primary cause or underlying comorbidity in children with severe pneumonia in high TB burden settings. Pneumonia case management guidelines should highlight clinical features that suggest TB in children with pneumonia, particularly a recent contact history, treatment failure and/or prolonged symptoms. HIV co-infection, malnutrition, and young age are recognised risk factors for TB as well as all-cause pneumonia. However, clinical features do not reliably predict or exclude TB in children presenting with severe pneumonia. Most studies identified in this review were from the African region and were in young children. The relationships identified between TB and pneumonia may be as relevant across a wider geography and populations, particularly in high-burden TB countries in the Asia-Pacific region. In TB endemic settings, effective approaches for early detection and treatment of TB in children and adolescents are needed to support pneumonia case-management.

## Additional material


Online Supplementary Document
