# Supplementary material for: A systematic review of clinical, epidemiological and demographic predictors of tuberculosis in children with pneumonia
Source: J Glob Health. 2022 Aug 9;12:10010. doi: 10.7189/jogh.12.10010 (PMC9527007; doi:10.7189/jogh.12.10010)
Supplement: Online Supplementary Document [file jogh-12-10010-s001.pdf]

Supplemental material

Appendix S1: MEDLINE (Ovid) search strategy

1. exp *Pneumonia/
2. ((respiratory adj3 infection*) or pneumonia or pneumonias or lung-inflammation* or lobitis or nonspecificinflammatory-lung-disease* or peripneumonia or pleuropneumonia or pleuropneumonitis or pneumonic-lung* or pneumonic-pleurisy or pneumonic-pleuritis or pneumonitides or pneumonitis or pulmonal-inflammation* or pulmonary-inflammation* or pulmonic-inflammation* or bronchiolitis).tw,kf.
3. *Pneumococcal Infections/
4. 1 or 2 or 3 5. exp Comorbidity/
6. co.fs.
7. hospitalization/ or patient admission/
8. adolescent, hospitalized/ or child, hospitalized/ or inpatients/
9. "Severity of Illness Index"/
10. (inpatient* or admission* or hospital* or severe or comorbidit* or co-morbidit* or multi-morbidit* or complication*).tw,kf.
11. 5 or 6 or 7 or 8 or 9 or 10
12. exp *Tuberculosis/
13. *Mycobacterium tuberculosis/
14. (Tuberculosis or tuberculous-infection* or tuberculous-lesion*).tw,kf. 15. 12 or 13 or 14
16. ep.fs.
17. incidence/ or prevalence/ or Prospective Studies/
18. *risk/ or exp *risk assessment/ or *risk factors/
19. (burden or risk or risks or determinant* or predict* or prospective* or incidence or prevalence).tw,kf.
20. exp "Sensitivity and Specificity"/
21. "cost of illness"/
22. 16 or 17 or 18 or 19 or 20 or 21
23. (infan* or toddler* or pre-schooler* or preschooler* or kinder or kinders or kindergarten* or kinder-aged or boy or boys or girl or girls or child or children or childhood or pediatric* or paediatric* or school-age* or schoolage* or schoolchild* or schoolgirl* or schoolboy* or adolescen* or youth or youths or teen or teens or teenage*).af.
24. (Xpert-MTBRIF or Xpert-MTB-RIF).tw,kf.
25. (x-ray* or xray* or imaging or radiolog* or cxr or radiogra*).tw,kf,hw.
26. (mantoux or tb-test* or skin-test* or blood or serolog* or sputum or stool or gastric-aspirate* or tuberculintest* or urine or lung or culture* or specimen* or PCR or polymerase-chain-reaction* or pleural-fluid* or NP-OP or swab* or nasopharyngeal-and-oropharyngeal or naso-pharyngeal-and-oro-pharyngeal or nasopharyngealoropharyngeal or naso-pharyngeal-oro-pharyngeal).tw,kf,hw.
27. Molecular Diagnostic Techniques/ 28. Tuberculin Test/
29. ip.fs.
30. (clinical-presentation* or clinical-feature* or clinical-sign* or clinical-symptom* or clinical-syndrome*).tw,kf.
31. (diagnos* or detection or investigation*).tw,kf.
32. early diagnosis/ or delayed diagnosis/
33. 24 or 25 or 26 or 27 or 28 or 29 or 30 or 31 or 32
34. 4 and 11 and 15 and 22 and 23 and 33
35. limit 34 to (english language and yr="1990 -Current")

Appendix S2: Prevalence of bacteriologically confirmed TB (BCTB) in studies of children with pneumonia

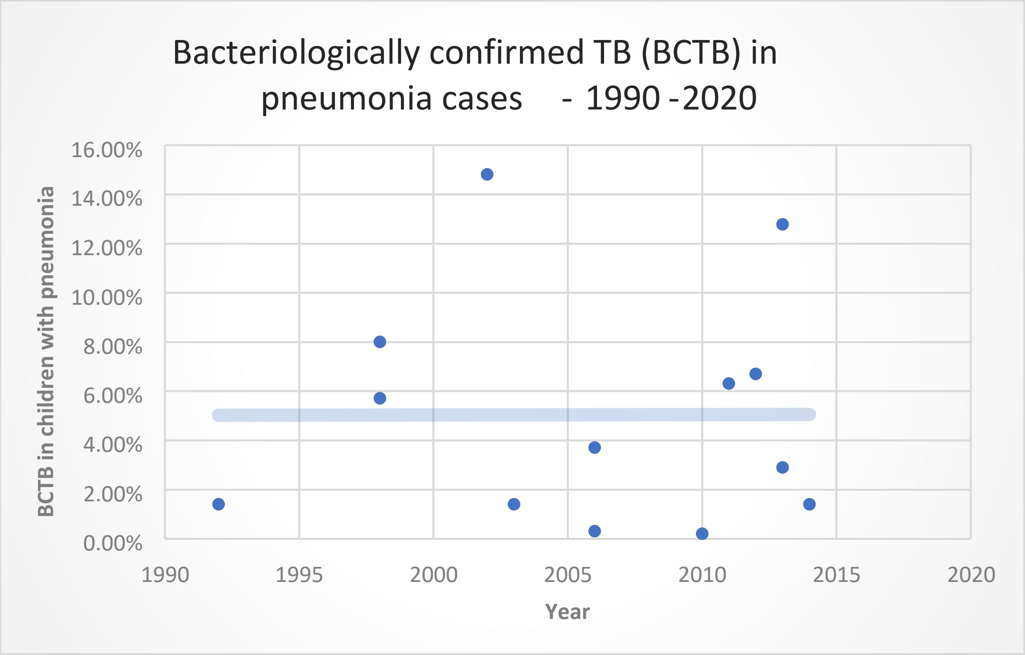

Appendix S3: EPHPP Quality Assessment Tool for Quantitative Studies, assessed at outcome level

Study	Selection Bias	Study design	Confounders	Blinding	Data collection methods	Withdrawals and dropouts	GLOBAL RATING
**Adegbola 1994**	Weak	Moderate	Moderate	Moderate	Weak-Moderate	Strong	Moderate
**Bolursaz 2017**	Weak	Moderate	Weak	Weak	Moderate	Moderate	Weak
**Chintu 2002**	Weak	Moderate	Moderate	Strong	Moderate	Moderate	Moderate
**Chisti 2015**	Moderate	Moderate-strong	Moderate	Weak	Moderate	Strong	Moderate
**Graham 2011**	Moderate	Moderate	Weak	Weak	Weak	Strong	Weak
**Hammitt 2012**	Moderate	Moderate	Weak	Moderate	Moderate	Moderate	Moderate
**Madhi 2000**	Weak	Moderate	Weak	Moderate	Moderate	Strong	Weak
**McNally 2007**	Moderate	Moderate	Strong	Moderate	Moderate	Strong	Strong
**Moore 2010**	Weak	Moderate	Moderate	Moderate	Weak	Strong	Weak
**Moore 2017**	Moderate	Moderate	Moderate	Moderate	Moderate	Moderate	Moderate
**Nantongo 2013**	Moderate	Moderate	Strong	Moderate	Moderate	Moderate	Strong
**O'Brien 2019**	Moderate	Moderate	Strong	Strong	Moderate	Strong	Strong
**Rennert 2002**	Weak	Moderate	Moderate	Moderate	Moderate	Strong	Moderate
**Uriyo 2006**	Moderate	Moderate	Weak	Moderate	Weak	Moderate	Weak
**Zar 2001**	Moderate	Moderate	Moderate	Moderate	Moderate	Strong	Strong
